# Lattice dynamics and elasticity for ε-plutonium

**DOI:** 10.1038/s41598-017-01034-6

**Published:** 2017-04-25

**Authors:** Per Söderlind

**Affiliations:** Lawrence Livermore National Laboratory, Livermore, CA 94550 USA

## Abstract

Lattice dynamics and elasticity for the high-temperature ε phase (body-centered cubic; bcc) of plutonium is predicted utilizing first-principles electronic structure coupled with a self-consistent phonon method that takes phonon-phonon interaction and strong anharmonicity into account. These predictions establish the first sensible lattice-dynamics and elasticity data on ε-Pu. The atomic forces required for the phonon scheme are highly accurate and derived from the total energies obtained from relativistic and parameter-free density-functional theory. The results appear reasonable but no data exist to compare with except those from dynamical mean-field theory that suggest ε-plutonium is mechanically unstable. Fundamental knowledge and understanding of the high-temperature bcc phase, that is generally present in all actinide metals before melting, is critically important for a proper interpretation of the phase diagram as well as practical modeling of high-temperature properties.

## Introduction

Plutonium metal remains one of the least understood, most controversial, and complex elemental metals in the periodic table of elements^1^. Consequently, great efforts have focused on this material from both theoretical and experimental angles. A few recent examples of experimental studies include x-ray spectroscopy^2^, neutron scattering^3^, and resonant ultra-sound spectroscopy (RUS)^4^. We shall also mention earlier inelastic x-ray measurements of phonon dispersions for the δ phase^5^ that were imperative for evaluating theory^6^.

On the theoretical side, fundamental modeling of plutonium metal has essentially taken two paths that suggest contrasting views on the nature of its 5*f* electrons. On one hand, these electrons are strongly correlated and well localized with dynamic self-energy fluctuations while on the other, the 5*f* electrons are more delocalized but are correlated via spin and orbital interactions. Both approaches have reported considerable success in describing some behaviors of plutonium. The strongly correlated electron assumption has mostly been modeled within dynamical mean-field theory (DMFT)^3, 6–12^ while weaker (intermediate) electron correlations have been examined by relativistic density-functional theory (DFT)^13–17^.

An effective alternative approach for δ-plutonium was proposed by Eriksson and coworkers^18, 19^ that introduced a superposition of localized and delocalized 5*f* states in their constrained model that produces remarkable agreement with measured photoemission spectra.

**Figure 1:**
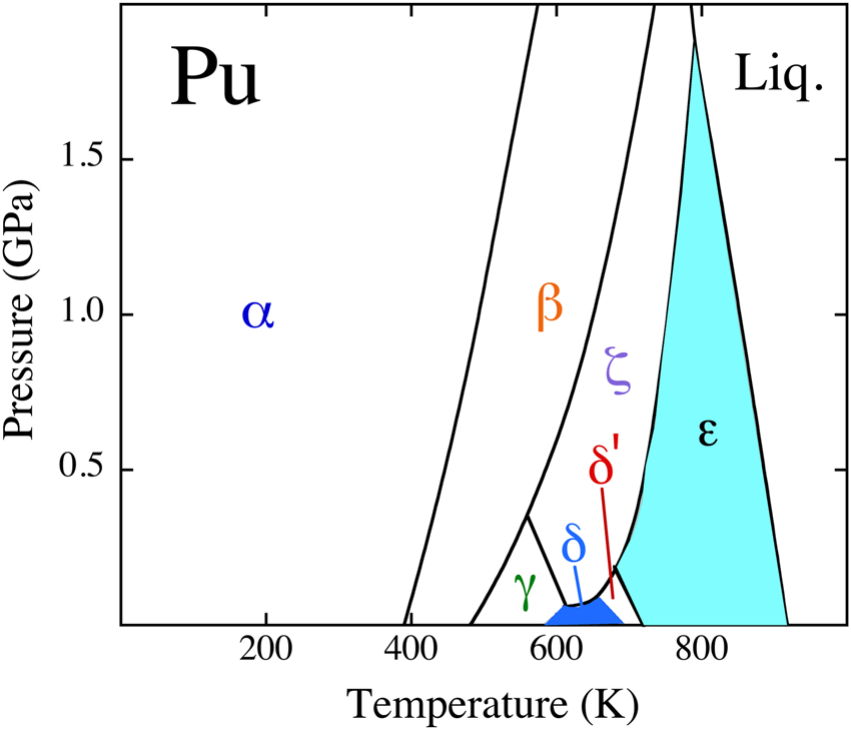
The experimental phase diagram for plutonium metal, redrawn after^20^.

The DMFT has primarily been employed for the low-density and high-temperature face-centered cubic (fcc) phase. Certainly, the properties of δ-Pu reflect much of the mystery of plutonium, but the phase diagram indicates many phases before melting at atmospheric pressure, see Fig. 1 ^20^. Density-functional theory, however, has been applied^13^ for all plutonium phases and reproduces the fundamental features of this very counterintuitive phase diagram except that the energy for ε-Pu appears too high relative to the other phases.

**Figure 2:**
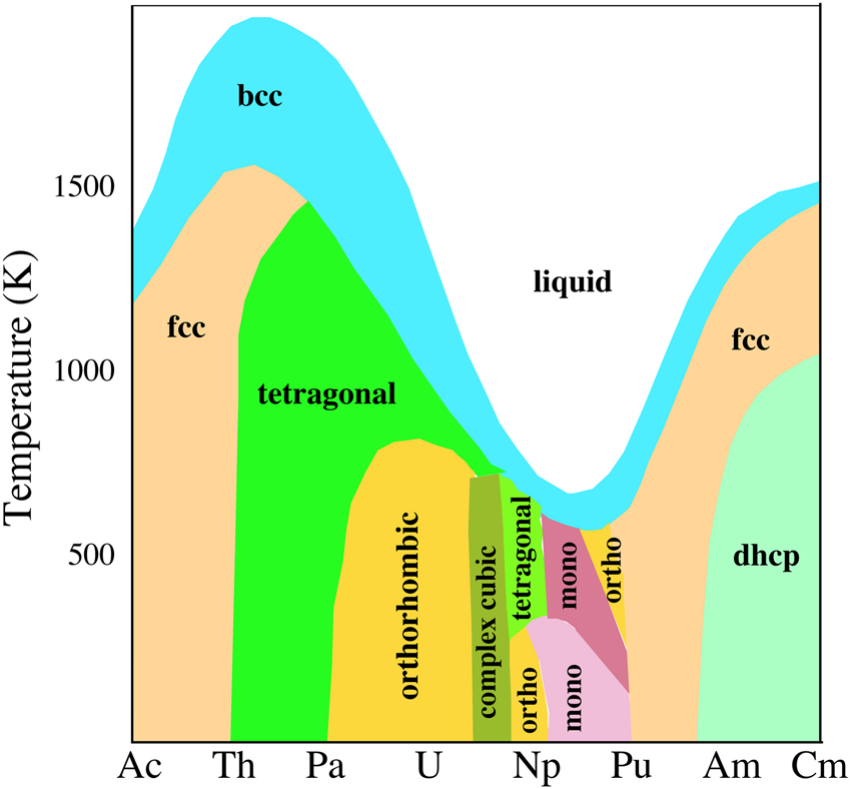
The actinides phase diagram, schematically redrawn after^23^.

It was recognized some time ago that theory encounters even more serious difficulties with ε-plutonium. Both DMFT^6^ and DFT^21^ failed to establish fundamental mechanical stability and consequently the suitability of the electronic structure of these techniques were questioned. On a more practical level these theories have thus not been able to accurately model thermodynamics that are required for nuclear-fuel simulations^22^, for example. Experimental efforts on plutonium have not helped either because ε-plutonium exists only at high temperatures making measurements troublesome to say the least. Consequently, elastic or phonon properties have never been measured accurately. In fact, very little information at all on ε-Pu can be found in the literature. Hence, remarkable as it may seem, almost nothing is known about this phase despite its dominance in the plutonium phase diagram at elevated temperatures, see Fig. 1. There has been voluminous research on δ-Pu, as outlined here, but a meager quantity on ε-Pu although it encompasses an order of magnitude larger space, see Fig. 1, in the low-pressure phase diagram. Actually, bcc is a very important phase more generally because it is prevalent in the phase diagram^23^ of all the actinide metals, see Fig. 2. Any progress in explaining this phase for plutonium is thus very helpful for a better understanding of the entire series of actinide metals.

In this Report, we address ε-plutonium for the first time in a realistic way. We apply a theoretical approach that uncovers the reason for its stability and existence while at the same time providing practically useful information and constraints for thermodynamic, equation of state, and strength modeling.

## Results

**Figure 3:**
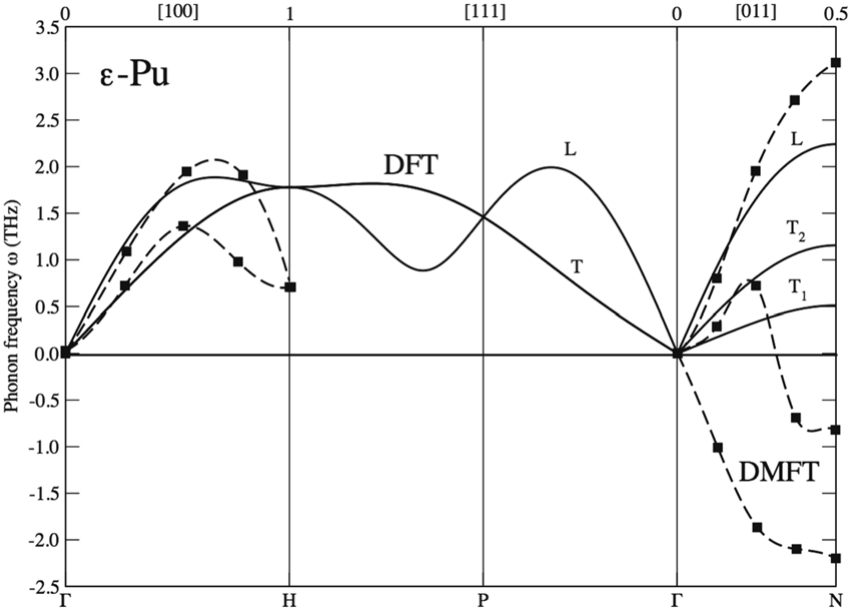
Calculated DFT (T = 900 K) phonons for ε-plutonium (full line) at the atomic volume 24.6 Å^3^ and DMFT results^6^ (dashed line).

**Figure 4:**
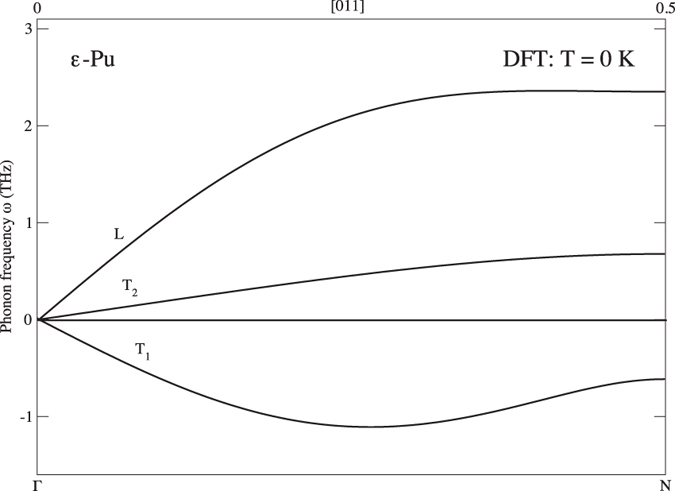
Calculated DFT (T = 0 K) *Γ-N* phonon branches for ε-plutonium at the atomic volume 24.6 Å^3^.

In Fig. 3 we show our main results of this Report, namely, the phonon structure for ε-plutonium calculated at ~900 K and 24.6 Å^3^. For comparison, we also include the DMFT results (dashed line) from Dai *et al*.^6^ in this figure. Our DFT and self-consistent phonons show no sign of instability related to the negative *C’* that is predicted at zero temperature, see our DFT (T = 0 K) result in Fig. 4 where unstable imaginary phonons are plotted as having negative frequencies. Contrarily, the DMFT results shown in Fig. 3 do not reproduce the mechanical stability and instead predict strongly imaginary transverse phonons in the *Γ-N* direction, similar to the DFT (T = 0 K) result in Fig. 4. Apparently, the strong electron correlations assumed in the DMFT model are not driving the stability and existence of ε-Pu.

One more important result of our study is the prediction of the elastic behavior. The calculated phonon dispersions in Fig. 3 define the single-crystal elastic moduli from the slopes approaching the *Γ* point and the results are summarized in Tables 1 and 2.

**Table 1:** Calculated elastic constants for ε-plutonium at an atomic volume of 24.6 Å^3^ and a temperature of 900 K.

*C*_*11*_	*C*_*12*_	*C*_*44*_	*C’*
50.1	45.8	12.7	2.2

*C*’ = ½ (*C*_11_ − *C*_12_) and all moduli are in units of GPa.

**Table 2:** Calculated Voigt averages of bulk and shear moduli *B*_*V*_ = (*C*_*11*_ + *2C*_*12*_)*/3* and *G*_*V*_ = (*C*_*11*_ − *C*_*12*_ + *3C*_*44*_)*/5* and *ĉ*_*11*_ = *B*_*V*_ + 4*G*_*V*_ */3* for ε-Pu together with corresponding theoretical and resonant ultra-sound spectroscopy^25^ data for δ-Pu.

Phase	Method	*B*_*V*_	*G*_*V*_	*ĉ*_*11*_
ε	Theory	47.2	8.5	58.5
δ	Theory	44.4	30.0	85.9
δ	RUS	29.7	21.0	51.3

All moduli are in the units of GPa.

In Table 1 we notice that *C*_*11*_ and *C*_*12*_ have nearly the same value which implies that the tetragonal shear constant *C’* is very small (~2.2 GPa). This anomalously small value (for aluminum it is an order of magnitude larger; *C*’ = 23 GPa) is consistent with the single-crystal measurement for δ-Pu (~5 GPa)^24^. Another similarity between ε and δ is that the anisotropy ratio *C*_*44*_ */C’* is very large in our model and that has also been observed experimentally^24^ for δ-Pu (5.8 and 7.0).

In order to make contact with possible future polycrystal RUS measurements we evaluate the Voigt averages of the elastic moduli for ε-Pu in Table 2. Here our ε-Pu data are compared with theory and RUS^25^ for δ-Pu. Because δ (fcc) and ε (bcc) are two different structures we do not expect the elastic moduli to necessarily be similar except for the bulk modulus that represents a hydrostatic strain that is less sensitive to the details of the crystal structure. Indeed, the calculated bulk moduli for ε-Pu and δ-Pu are almost the same; see Table 2.

**Figure 5:**
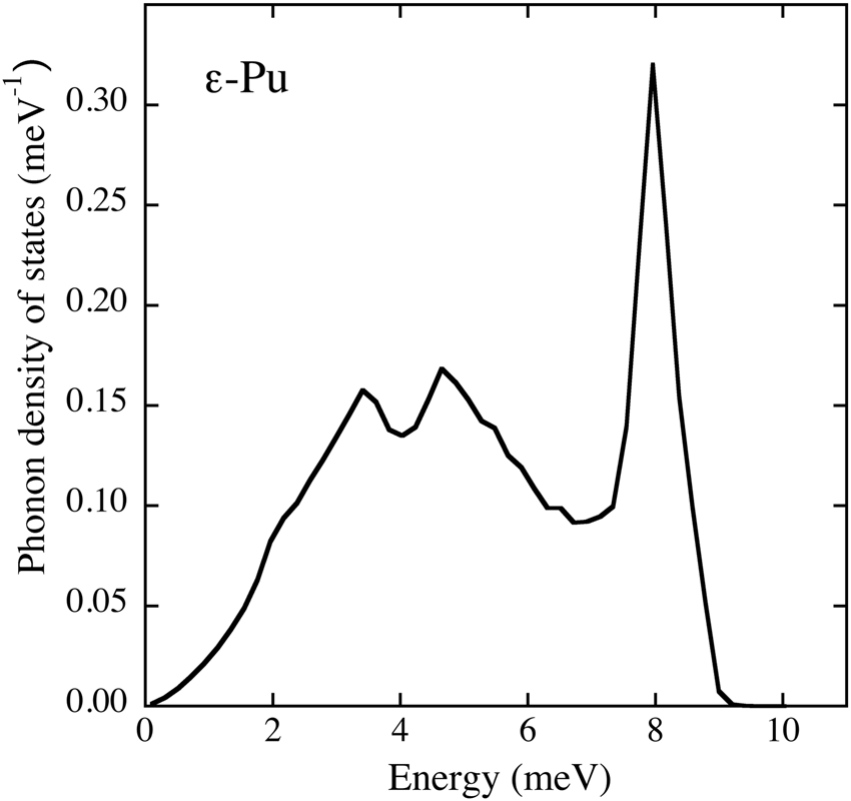
Calculated (T = 900 K) phonon density of states for ε-plutonium at the atomic volume 24.6 Å^3^.

In addition to the phonon dispersions we predict the phonon density of states (DOS) shown in Fig. 5. The shape of the phonon DOS resembles that of γ-U^26, 27^ but no measured data for ε-Pu are available for a direct comparison. The phonon DOS is of course important for thermodynamical models that depend on characteristic phonon temperatures, *θ*_*n*_, that Wallace^28^ defines as:

1 $${\rm{ln}}(k{\theta }_{0})={\langle{\rm{ln}}(\hslash \omega )\rangle}_{BZ}$$

2 $$k{\theta }_{1}=4/3{\langle\hslash \omega \rangle}_{BZ}$$

3 $$k{\theta }_{2}={(5/3{\langle{(\hslash \omega )}^{2}\rangle}_{BZ})}^{1/2}$$

where <…>_*BZ*_ indicates a Brillouin zone average of the phonon frequencies (*ω*) while *k* and *ħ* are the Boltzmann’s and Dirac’s constants, respectively. For ε-plutonium we determine the Debye temperatures *θ*_*0*_, *θ*_*1*_, and *θ*_*2*_ from the phonon DOS (Fig. 5) and they are 54, 82, and 86 K, respectively. These numbers are close but tolerably higher than those estimated for ε-Pu in Wallace’s analysis (45, 63, and 63 K)^28^ but smaller than a value (105 K) obtained from a simple Debye-Grüneisen quasi-harmonic model^29^ that utilizes the zero-temperature bulk modulus. Incidentally, a recent resonant ultra-sound spectroscopy study by Suzuki *et al*.^30^ reports a Debye temperature for δ-Pu of 91 K that is close to our calculated *θ*_*2*_ for ε-Pu. No measured Debye temperatures have been disclosed for ε-Pu, however.

## Discussion

We have calculated lattice dynamics, elasticity, and Debye temperatures for an important phase of plutonium, namely the bcc ε phase. Neither theory nor experiments have been able to deduce these quantities for ε-Pu until now. Elastic and vibrational behaviors are very important because they are necessary components of any thermodynamics, equation of state, or strength model. Our data are particularly significant because experimental data that could be used for such modeling are not available.

A comparison with DMFT reveals that strong 5*f* electron correlation is not a driver for the formation of ε-Pu at high temperatures. On the contrary, it is formed because of strong anharmonic effects and entropy^31^ that are properly accounted for by phonon-phonon interactions determined from our self-consistent phonons and DFT electronic structure.

With the present predictions for ε-Pu the intermediate 5*f*-electron correlation picture outlined by our DFT model has proven to successfully explain all known phases of plutonium metal. Furthermore, the results are meaningful for plutonium modeling and may serve as benchmarks or constraints for less accurate or semi-empirical methods.

## Methods

In recent years quantum molecular dynamics (QMD)^32^ has been pursued to describe high-temperature phases in materials but for plutonium this is very difficult because of the 5*f*-electron correlations that require special care for the magnetic and relativistic interactions. Also, QMD for plutonium demands computational resources that are far beyond current capabilities.

Instead we calculate highly accurate relativistic spin- and orbital-polarized DFT-type electronic structures and total energies that we couple with an efficient self-consistent phonon scheme developed by Souvatzis and coworkers^33, 34^. The phonon method^34^ uses forces on atoms that are thermally displaced from their ideal positions in the crystal and these are obtained from computations of the plutonium electronic structure and total energy. The forces are obtained numerically from total-energy responses to very small atomic displacements (see below). This procedure coupled with the iterative nature of the phonon scheme^34^ adds 2–3 orders of magnitude to the computational effort compared to a conventional zero-temperature phonon calculation. Still, it is far more efficient than a full-fledged QMD simulation and it accounts for decisive anharmonic contributions to the phonons.

The DFT electronic structure is constructed from a full-potential linear muffin-tin orbitals method^35^ that is fully relativistic but also includes an orbital polarization contribution^36^ derived from atomic physics that is known to be an important perturbation of the 5*f*-electron states of plutonium^37, 38^. The generalized gradient approximation is assumed for the electron exchange and correlation interactions. No parameters are adjustable; the (Racha) parameters associated with the orbital polarization are small (~50–60 mRy for the 5*f* electrons) and calculated self-consistently from Slater integrals of wave functions and the electronic structure of ε-Pu is fundamentally treated the same way as for all other phases of plutonium^13^.

In this approach, the total energy is the most robust quantity and all presented results are derived from it while other calculated physical properties are perhaps less reliable. Our electronic-structure model for ε-plutonium resides above the magnetic ordering temperature and treated as paramagnetic with disordered magnetic moments. This is a reasonable static approximation of the fluctuating spin state that has been proposed for δ-Pu^4^ and likely persists into the ε phase. For this purpose, we apply a special quasi-random structure (SQS)^39^ containing 16 atoms where the spin up and spin down are distributed on atoms in equal proportion to best approximate a disordered or paramagnetic configuration.

From the ε-Pu electronic structure and total energy we compute the atomic forces by introducing very small displacements (±~0.01 Å) on each atom (for each *x*, *y*, and *z* coordinate). A polynomial is adapted to the total energies and the force components extracted from analytical differentiation of these polynomials. This is the most accurate way to calculate atomic forces. There is no need to try and compute less accurate Hellman-Feynman forces that are numerically difficult to obtain correctly and also not well defined when substantial spin-orbit interaction and orbital polarization, as is the case here, are perturbing the electronic structure.

For the temperature-dependent phonon method^34^ a bcc super-cell is necessary and for computational expedience a 27-atom super-cell is utilized that compares rather well with a larger 64-atom cell in limited convergence tests. In this 27-atom super-cell the spins are aligned in an anti-ferromagnetic (AF) fashion (13 and 14 spin up and down, respectively) in lieu of the disordered configuration for entirely practical reasons. However, this technical simplification is justified because the energetics of the bcc instability is the same for the AF-ordered and the disordered spin configuration.

**Figure 6:**
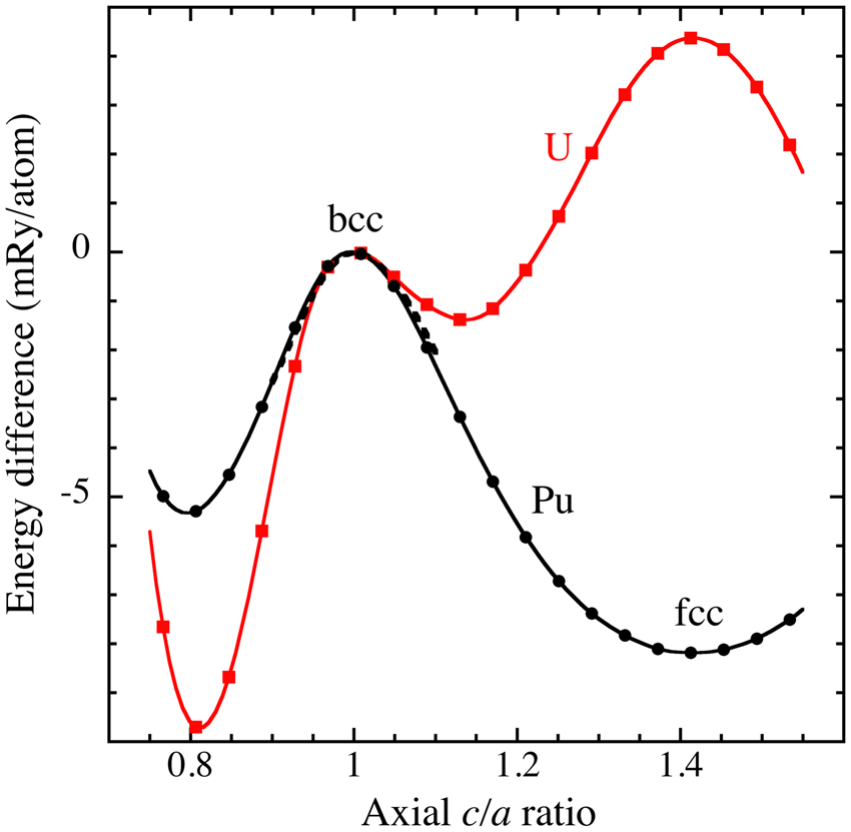
Calculated zero-temperature energy difference, relative to the bcc phase (paramagnetic, see main text), as a function of axial *c/a* ratio for uranium and plutonium. The dashed curve shows results for anti-ferromagnetic plutonium.

In Fig. 6 we show the energies as functions of axial *c/a* ratio in the tetragonal body-centered cubic cell for uranium and plutonium (AF and disordered spin moments). The atomic volumes are kept fixed to their theoretical equilibrium volumes of 20.7 and 24.6 Å^3^, respectively, but small changes of these volumes only weakly influence these so-called Bain-transformation curves. In the plot, *c/a* = 1 and *c/a* = 1.414, correspond to the bcc and fcc crystals, respectively. Notice that for both metals the energy shows a local maximum for the bcc phase thus indicating a negative elastic constant, *C’*, and mechanically instability. An obvious consequence of a negative *C’* is that a transverse mode of the *Γ-N* branch must be imaginary, see our (T = 0 K) calculation of this branch in Fig. 4. Interestingly, the instability has similar magnitude for uranium and plutonium (similar curvature at *c/a* = 1). This is an important observation because for bcc uranium (γ-U) strong anharmonic contributions due to phonon-phonon coupling produce very good phonon density of states^27^ and the aforementioned similarity between U and Pu then suggest it may be true also for ε-plutonium. The phonon density of states for ε-plutonium is shown in Fig. 5.

In regards to our simplification to align spins anti-ferromagnetically for the phonon calculation, we show in Fig. 6 the dashed line assuming the lowest-energy AF state (*L*1_0_; CuAu structure for *c/a* = 1.414) and the result is essentially identical to that of the magnetic disorder (16-atom SQS cell). This means that both spin configurations should give comparable phonons, at least close to the instability. Figure 6 further shows that plutonium has a positive but very small *C’* in the fcc (δ) phase in agreement with single-crystal experiments and it may therefore be no surprise that our electronic-structure model reproduces the experimental δ-Pu phonon dispersions quite well^16^.

We deduce the elastic properties of ε-plutonium (Tables 1 and 2) from the phonon dispersions approaching the *Γ* point. There are more such phonons than elastic moduli for a cubic system (3) and thus one obtains an over-determined system of linear equations when evaluating the elastic constants. We find a unique solution to this system of equations by minimizing the least-square residual error.
